# Supplementary material for: Developmental Regulation and Functional Prediction of microRNAs in an Expanded Fasciola hepatica miRNome
Source: Front Cell Infect Microbiol. 2022 Feb 10;12:811123. doi: 10.3389/fcimb.2022.811123 (PMC8867070; doi:10.3389/fcimb.2022.811123)
Supplement: Supplementary file 1 [file DataSheet_1.docx]

# SUPPLEMENTARY DATASHEET 1: Mature consensus sequences in the Fasciola hepatica miRNome

>fhe-novelmir-31-3p

attagaactggtgggcactt

>fhe-novelmir-31-5p

gttctaccatcgtaatatgca

>fhe-novelmir-32-5p

atcccaagctcagagtactg

>fhe-novelmir-33-3p

tttttccggctgctaaaatct

>fhe-novelmir-34-3p

taaccaaaacgttggtgact

>fhe-novelmir-35-5p

aggatccgggcgaagacatcc

>fhe-novelmir-36-5p

taatcagtcgacttttcggtca

>fhe-novelmir-37-5p

gaatgacggatttatcggagt

>fhe-novelmir-38-5p

tacgcgctccttaagatgctgat

>fhe-mir-11586-3p

gtaagacgatcgtagttgacg

>fhe-novelmir-39-5p

tcctccacacatcgttgacg

>fhe-novelmir-4-5p

gatggacaaactatgcagacg

>fhe-novelmir-40-3p

gttgttcgactgacgatcgttc

>fhe-novelmir-40-5p

atggttgtaagtcgaacaact

>fhe-novelmir-41-5p

tttatgtccatggtgtagtct

>fhe-novelmir-42-5p

atggcgaatgtgagaaccatg

>fhe-novelmir-43-3p

gacgatgatgactgacacactgaat

>fhe-novelmir-44-5p

actggactgacttttccgaga

>fhe-novelmir-45-3p

ttggaaaagtaggttgtgcct

>fhe-mir-11587

ttccggcagcttagtacagct

>fhe-novelmir-5-5p

tgaccgtaccactgagtgaccc

>fhe-novelmir-6-5p

ttagtgcattgttgatagacc

>fhe-novelmir-7-5p

aacacagcgtagtcgaaatc

>fhe-novelmir-8-3p

gtacttactcataacatgcc

>fhe-novelmir-9-3p

aaggattcggagatagttaag

>fhe-pubnovelmir-1

aaacagctgtacagtgcttct

>fhe-mirna-novel-01-3p

atggatggatagatggatgg

>fhe-pubnovelmir-10

caagctcgggtatacaggagcag

>fhe-pubnovelmir-11

cagtcggtagagcatcagact

>fhe-pubnovelmir-12

cctcaacaagaaggctgttggatg

>fhe-mir-12-5p

tgagtatttcatcaagtagtga

>fhe-pubnovelmir-13

cgttgcaccgttcggaattcgggca

>fhe-mirna-novel-05-5p

gaccacttcacctacctcgg

>fhe-pubnovelmir-14

gaccggtggtggtcgagtgggtt

>fhe-mirna-novel-03-5p

gacggggtggccgagtggtta

>fhe-mirna-novel-15-5p

gagagagagagagagggagaga

>fhe-mirna-novel-06-3p

gaggtaggtgaagtggtcga

>fhe-pubnovelmir-2

accctcatttagatcgaaggt

>fhe-pubnovelmir-15

gaggtagtgagttgtatgtctg

>fhe-pubnovelmir-16

gatggagtagctatggggtct

>fhe-pubnovelmir-17

gcctccatagctcagtggtcaga

>the-mir-124-3p

taaggcacgcggtgaatgtca

>fhe-pubnovelmir-18

gggtggatacagtcggttatg

>fhe-pubnovelmir-19

gtcagcgaagacgtcgggaa

>fhe-pubnovelmir-20

gtggcctcgtagctcagctggtag

>fhe-pubnovelmir-21

tagagtacctgtagatttag

>fhe-pubnovelmir-22-3p

tagtcactgggctacgaacacg

>fhe-pubnovelmir-23-3p

tcagcaccggccgaaacgacac

>fhe-pubnovelmir-23-5p

tcagcaccggccgaaacgac

>fhe-pubnovelmir-24

tcagttctcattaggcatgacatg

>fhe-pubnovelmir-3

acgatgatgatgatgatgattt

>fhe-mirna-novel-10-3p

tccatcatcatcatcatcatcatc

>the-mir-125a-5p

tccctgagaccctagagtttcc

>fhe-pubnovelmir-25

tccccgtaatcgaactgttgt

>fhe-pubnovelmir-26

tccgaaaacgcgatggaacct

>fhe-pubnovelmir-27

tgatgatggatttactgttgt

>fhe-cin-mir-4006b-3p

tggaacaatgtaggtaaggg

>fhe-pubnovelmir-28

tggaagcactgtacagctgtttt

>fhe-pubnovelmir-29

tttgcatatctaagtcggaca

>fhe-pubnovelmir-4-3p

agacactcagaggacgatcagt

>fhe-pubnovelmir-4-5p

tgaccgtcacctgagtgtcc

>fhe-pubnovelmir-5

aggagggcaattttatgacttt

>fhe-pubnovelmir-6

aggctgtgtgtagagcaagttg

>fhe-mir-125b-3p

agcaattttctctctcaggtat

>fhe-pubnovelmir-7

agtggtgatggtcgagtggtttag

>fhe-pubnovelmir-8

atgaaacagctgtacagtgc

>fhe-pubnovelmir-9

atgagacggtgagtgatgaatt

>fhe-mir-125b-5p

cccctgagactgataattgctcc

>fhe-mir-133-3p

ttggtccctatcaaccagctat

>fhe-mir-184-5p

tggacggagatttgttaagagc

>fhe-mir-190-3p

ccagtgaccaaacatattctc

>fhe-bantam-3p

tgagatcgcgattaaagctggt

>fhe-mir-190-5p

agatatgtttgggttacttggtg

>fhe-mir-190b-5p

tgatatgtatggttttcggttg

>fhe-mir-1989-5p

tcagctgtgttcatgtcttcga

>fhe-mir-1992-3p

tcagcagttgcaccattgacg

>fhe-mir-1b-3p

tggaatgttgtgaagtatgtac

>fhe-mir-210-3p

ttgtgcgtcgtttcagtgaccgaa

>fhe-mir-2162-3p

tattatgcaacatttcactct

>fhe-mir-2162-5p

agtgggatggttgcatacga

>fhe-mir-219-5p

tgattgtccattcgcatttcttg

>fhe-mir-277-3p

taaatgcattttctggcccgtaa

>fhe-let-7-5p

agaggtagtgactcatatgact

>fhe-mir-277a-3p

aaaatgcatcatctacccgaga

>fhe-mir-278-3p

tcggtgggagtatcattcgtgc

>fhe-mir-281

tgtcatggagttgctctctaca

>fhe-mir-2a-3p

tcacagccagaattgatgaacg

>fhe-mir-2a-5p

cagtcaatactggctggaggca

>fhe-mir-2b-3p

tatcacagccctgcttgggacaca

>fhe-mir-2b-5p

cgtcctcgggggttgtgacaca

>fhe-mir-2c-3p

tatcacagccgtgcttaagggctt

>fhe-let-7a-5p

ggaggtagttcgttgtgtggt

>fhe-mir-2d-3p

tatcacagtcctgcttaggtgacga

>fhe-mir-2e-3p

tatcacagtccaagctttggtaaa

>fhe-mir-2e-5p

taccaacttagactgagttat

>fhe-mir-2f-3p

tcacagccaatattgatgcctg

>fhe-mir-307-3p

tcacaacctacttgattgagggg

>fhe-mir-31-3p

agctacaccatattttgcccacg

>fhe-mir-31-5p

tggcaagattatggcgaagctga

>fhe-mir-3479-3p

tattgcactttccttcgcctta

>fhe-mir-3479b-3p

gattgcactactcatagccttc

>fhe-mir-36a-3p

tcaccgggtagacattcattcac

>fhe-let-7b-5p

tgaggtagtaggttgtatagt

>fhe-mir-36b-3p

ccaccgggtagacattcatccgc

>fhe-mir-46-3p

tgtcatggagttgctctctaca

>fhe-mir-46-5p

aggagggcaattttatgacttt

>fhe-mir-61-3p

tgactagaaagtgcactcacttc

>fhe-mir-61-5p

tgtgagtctctttcttgtccatg

>fhe-mir-7-5p

tggaagactggtgatatgttgtt

>fhe-mir-71a-3p

tctccctaccccgtcttttctg

>fhe-mir-71a-5p

tgaaagacatgggtaatgaggt

>fhe-mir-71b-5p

tgaaagacgatggtagtgagatg

>fhe-mir-71c-5p

tgaaagacttgagtagtgagacg

>fhe-mir-1-3p

tggaatgtggcgaagtatggtct

>fhe-mir-745-3p

tgctgccttataagagctgtga

>fhe-mir-745-5p

tcagttctcattaggcatgac

>fhe-mir-745b-3p

aagctgccaagcgaagggccaag

>fhe-mir-750-3p

ccagatctgactcttccagctctt

>fhe-mir-755-3p

tgagattcaactacttcagctg

>fhe-mir-7a-5p

tggaagacttgtgattaagttgtt

>fhe-mir-8-3p

taatactgtttggtaaagatgcc

>fhe-mir-8437-3p

tggcgcttagttatatgtcatcg

>fhe-mir-87-3p

gtgagcaaagtttcaggtgtga

>fhe-mir-10-3p

aaattcgagtctacaaggaac

>fhe-mir-9-5p

tctttggttatcaagcagtatga

>fhe-mir-9389-5p

aggcgctttgattgtccacactga

>fhe-mir-96-5p

cttggcactttggaattgtcac

>fhe-novelmir-1-3p

tttatctcgtccaggtagacc

>fhe-novelmir-10-5p

tccccgatcaaagatgcaagc

>fhe-novelmir-11-5p

gctccaaaaccgctctgaaacc

>fhe-novelmir-12-3p

ttcaggatccgggcgaagac

>fhe-novelmir-13-3p

agatgttggctttagtatact

>fhe-novelmir-14-3p

ccaccgtcatcggttacttt

>fhe-novelmir-15-5p

tccccaccatcgtcggttact

>fhe-mir-10-5p

aaccctgtagacccgagtttgca

>fhe-novelmir-16-3p

gtggatgatgcacaccaaaaacc

>fhe-novelmir-17-5p

ttaccgatgacgccttttgct

>fhe-novelmir-18-5p

gaggtacattcgctaacgactga

>fhe-novelmir-19-5p

aaccggtgttggtcaggtgatt

>fhe-novelmir-2-3p

atcggcctttgagactatgggg

>fhe-novelmir-2-5p

aagtctcagggtttggttgc

>fhe-novelmir-20-5p

tatgaaaccggatgcaagacc

>fhe-novelmir-21-3p

tccatttatgtagacactgat

>fhe-novelmir-22-3p

ttcggagatagttaaggaact

>fhe-novelmir-23-5p

ttgactgtaccactgagtgacct

>fhe-mir-11584-3p

cattatataagattgaggctct

>fhe-novelmir-24-5p

ggaacacactctgatagctg

>fhe-novelmir-25-3p

aaagtgaacactggtggatgt

>fhe-novelmir-25-5p

atttatcggagttcaataaatg

>fhe-novelmir-26-3p

catcgtgggattagcttaat

>fhe-novelmir-27-5p

ttctatatatcgtggtaccg

>fhe-novelmir-28-3p

tagaactggtgggcactttcc

>fhe-novelmir-29-3p

taatcggaacgtcgacggact

>fhe-novelmir-29-5p

tcagtcgatgtttcggttacc

>fhe-novelmir-3-3p

ttagacctttttgtagcaact

>fhe-novelmir-30-3p

tcaagaagacggagtagcaatg

>fhe-mir-11585

accggtttcgtcgttcaacac
